# Supplementary material for: Temperature Control of Yellow Photoluminescence from SiO2-Coated ZnO Nanocrystals
Source: Nanomaterials (Basel). 2022 Sep 27;12(19):3368. doi: 10.3390/nano12193368 (PMC9565792; doi:10.3390/nano12193368)
Supplement: Supplementary file 1 [file nanomaterials-12-03368-s001.zip › nanomaterials-1850771-supplementary.pdf]

SUPPORTING INFORMATION

Temperature Control of Yellow Photoluminescence of $ZnO–SiO_2$ Nanorods

Narender Kumar,[1] Vijo Poulose, [1] Youssef Taiser Laz,[2] Falguni Chandra, [1] Salma Abubakar,[3] Abdalla S. Abdelhamid, [1] Ahmed Alzamly, [1] and Na'il Saleh*,[1]

[1] Department of Chemistry, College of Science, United Arab Emirates University, P.O. Box 15551, Al Ain, United Arab Emirates

[2]Academic Support Department, Abu Dhabi Polytechnic, Al Ain, United Arab Emirate

[3]Chemistry Program, New York University Abu Dhabi (NYUAD), Saadiyat Island, United Arab Emirates

TABLE OF CONTENTS

Part I: Material Characterizations of ZnO–SiO_2 Nanorods ………………………..… S3

FTIR Spectra ……………………..…………………………………..………..…….S3

XRD………………………..……………………………………………..…..……S4

Particle Size Distribution……………………………………………………………S5

Part II: Optical Characterization of ZnO–SiO_2 Nanorods …………………………..… S6

PL and PLE Measurements ………..……………………………………....… S6

TRPL measurements ……………………………..……………………………..……….S7

DAS measurements…………………………………………………………………..S8

Part I: Material Characterizations of $ZnO–SiO_2$ Nanorods

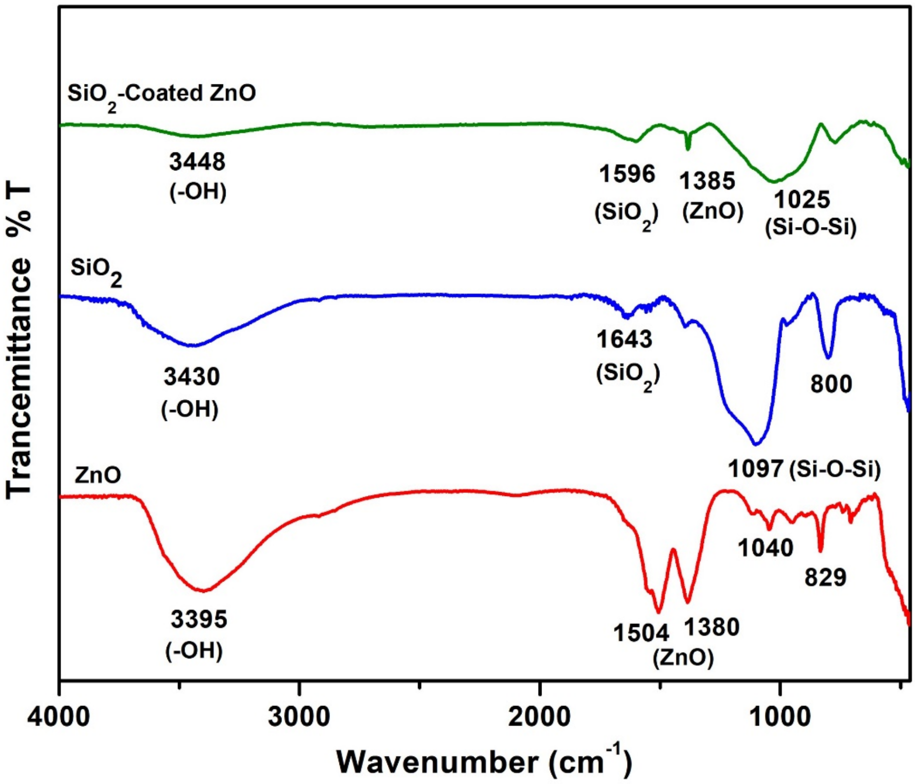

Figure S1. FTIR spectra for a) SiO_2-coated ZnO slides b) SiO_2 powders c) ZnO powders

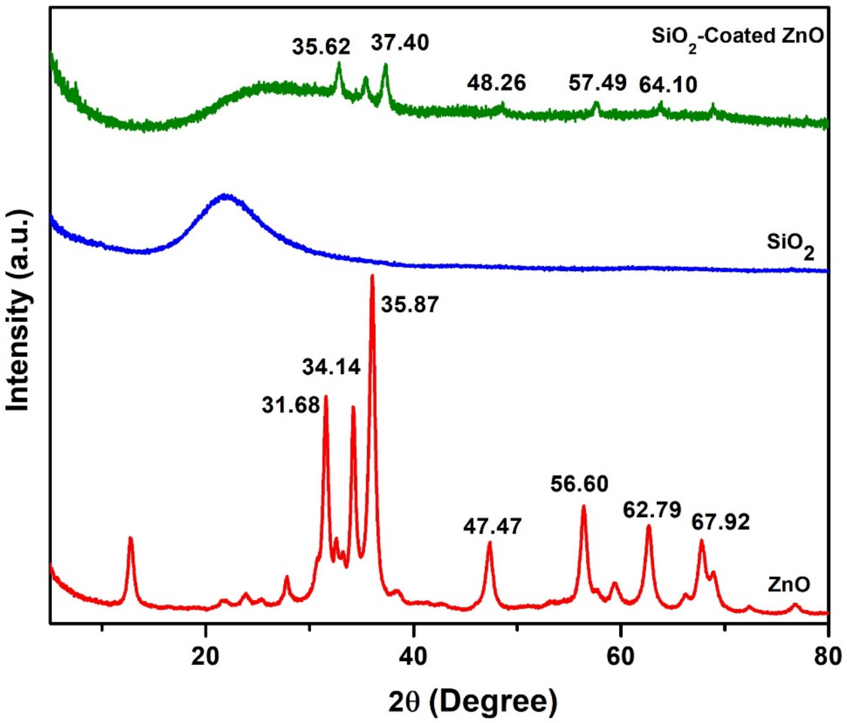

Figure S2. The powder X-ray diffraction pattern of (A) Silica, (B) ZnO (C) our sample.

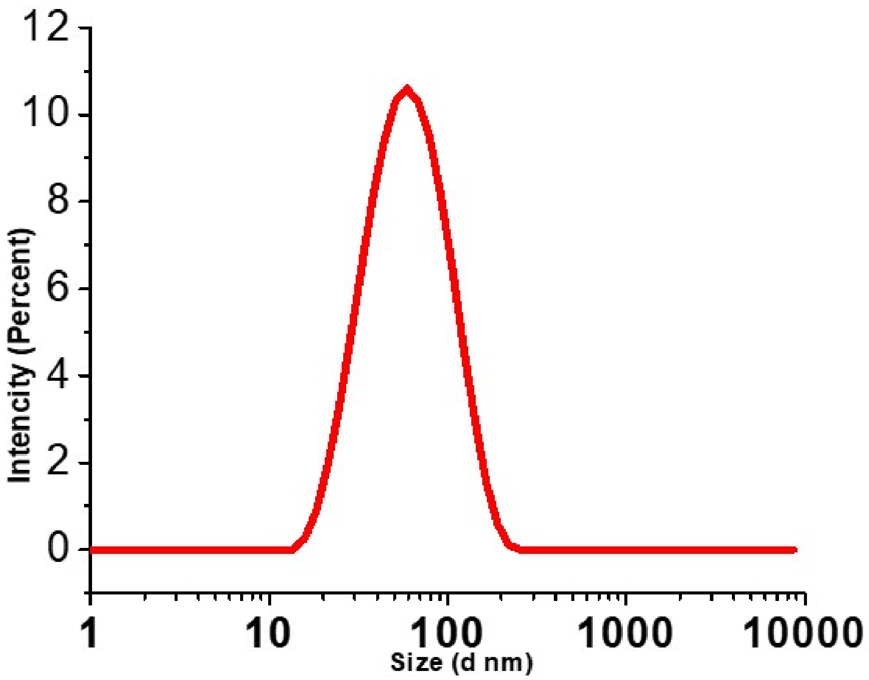

Figure S3. Particle size distribution of the sample.

Part II: Optical Characterizations of ZnO–SiO_2 Nanorods

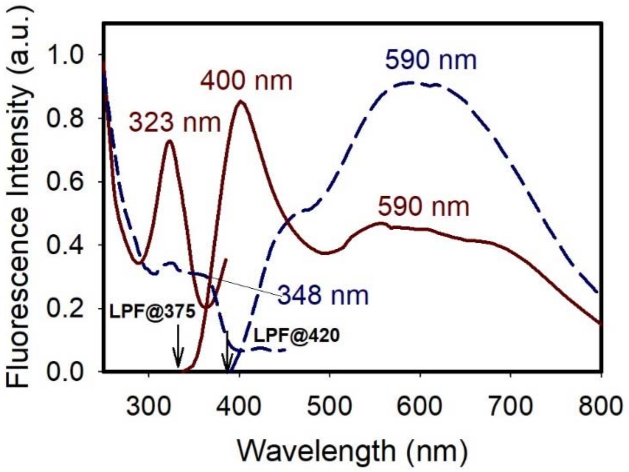

Figure S4. Photoluminescence (PL) and excitation spectra at 298 K upon excitation at 375 nm and monitoring at 550 nm (dashed lines, a); and upon excitation at 320 nm and monitoring at 411 nm (solid lines, b).

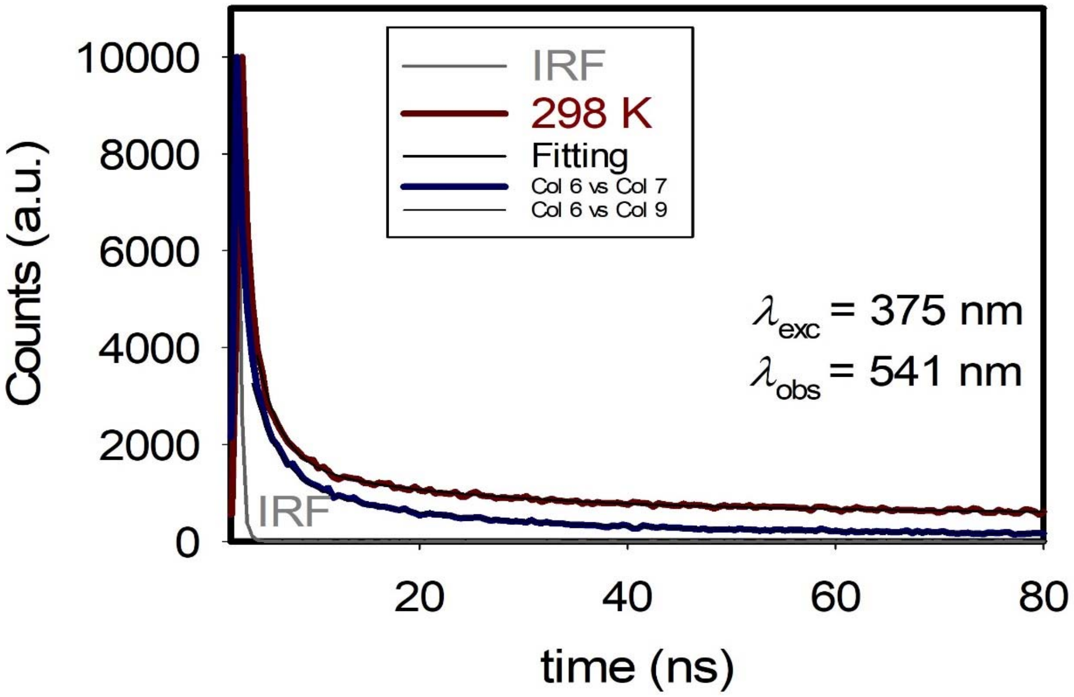

Figure S5. PL decays upon excitation at 375 nm monitored at the yellow band (590 nm) of $ZnO–SiO_2$ solids as a function of temperatures in kelvin (K).

Table S1. The observed excited-state lifetime for different $ZnO–SiO_2$ samples.

**Samples**	**λ_{obs} (nm)**	**τ_1 (ns)**	***f_1*%**	**τ_2 (ns)**	***f_2*%**	**τ_3 (ns)**	***f_3*%**	**$\tau_{average}$ (ns)**	**Chi-Square**
**298 K**	541	2.2	13	16.8	20	144.6	67	100	1.015
**378 K**	541	2.7	26	16.9	33	105.3	41	59	1.238

The time resolution was ~30 picoseconds and the excitation wavelength was 375 nm.

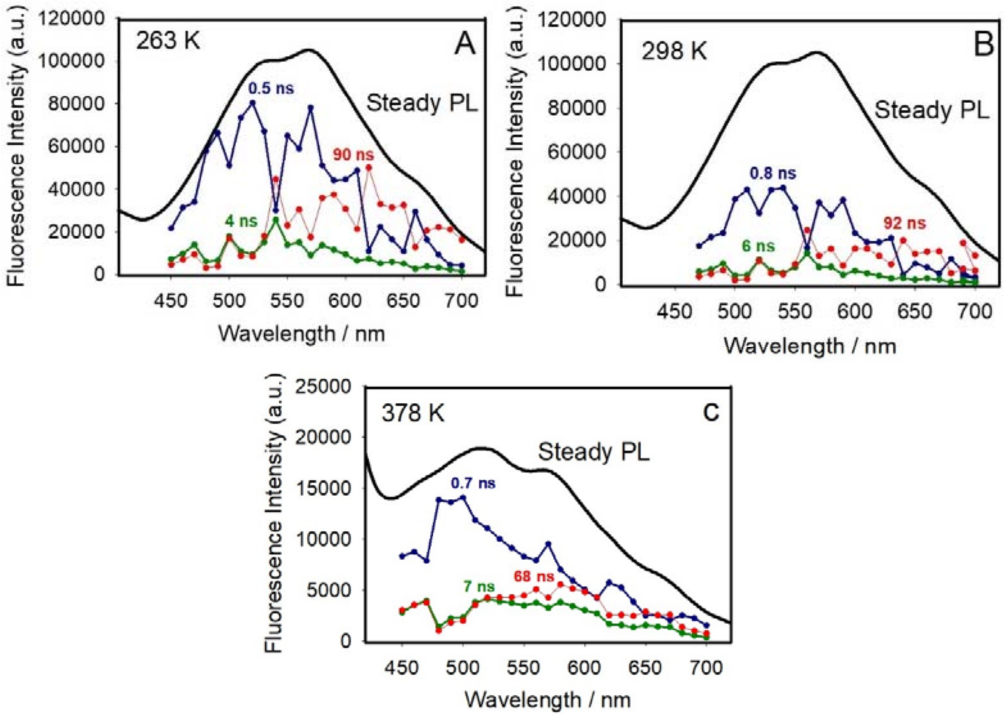

Figure S6. Decay-associated spectra (DAS) of a three-component mixture of exciton fluorophores for ZnO–SiO_2 solids at different temperatures upon excitation at 375 nm. The corresponding steady-state spectra of each solid are also shown for comparison (Experimental Section).
